# Effects of *Bacillus subtilis* A-5 and its fermented γ-polyglutamic acid on the rhizosphere bacterial community of Chinese cabbage

**DOI:** 10.3389/fmicb.2022.954489

**Published:** 2022-08-15

**Authors:** Naling Bai, Hanlin Zhang, Yu He, Juanqin Zhang, Xianqing Zheng, Haiyun Zhang, Yue Zhang, Weiguang Lv, Shuangxi Li

**Affiliations:** ^1^Eco-environmental Protection Research Institute, Shanghai Academy of Agricultural Sciences, Shanghai, China; ^2^Agricultural Environment and Farmland Conservation Experiment Station of Ministry Agriculture and Rural Affairs, Shanghai, China; ^3^Shanghai Key Laboratory of Horticultural Technology, Shanghai, China; ^4^Key Laboratory of Low-carbon Green Agriculture, Ministry of Agriculture and Rural Affairs, Shanghai, China

**Keywords:** rhizosphere, γ-polyglutamic acid, *Bacillus subtilis*, bacterial community, high-throughput sequencing

## Abstract

Chemical fertilizer reduction combined with novel and green agricultural inputs has become an important practice to improve microecological health in agricultural production. Given the close linkages between rhizosphere processes and plant nutrition and productivity, understanding how fertilization impacts this critical zone is highly important for optimizing plant–soil interactions and crop fitness for agricultural sustainability. Here, by using a pot experimental system, we demonstrated that nitrogen fertilizer reduction and microbial agent application promoted plant fitness and altered the microbial community structure in the rhizosphere soil with the following treatments: no fertilization, CK; conventional chemical fertilizer, CF; 30% reduced nitrogen fertilizer, N; 30% reduced nitrogen fertilizer with pure γ-PGA, PGA; 30% reduced nitrogen fertilizer with *Bacillus subtilis* A-5, A5; 30% reduced nitrogen fertilizer with γ-PGA fermentation broth, FJY. The PGA, A5, and FJY treatments all significantly promoted crop growth, and the FJY treatment showed the strongest positive effect on Chinese cabbage yield (26,385.09 kg/hm^2^) (*P* < 0.05). Microbial agents affected the α diversity of the rhizosphere bacterial community; the addition of *B*. *subtilis* A-5 (A5 and FJY treatments) significantly affected rhizospheric bacterial community structure. Urease activity and soil pH were the key factors affecting bacterial community structure and composition. The FJY treatment seemed to influence the relative abundances of important bacterial taxa related to metabolite degradation, predation, and nitrogen cycling. This discovery provides insight into the mechanism underlying the effects of microbial agent inputs on rhizosphere microbial community assembly and highlights a promising direction for the manipulation of the rhizosphere microbiome to yield beneficial outcomes.

## Introduction

Microorganisms constitute the core components of the soil ecosystem, occupying dominant positions in the processes of nutrient cycling and energy transformation (Wardle et al., 2004; Li et al., 2013). Interactions between plant roots and soil microorganisms are critical for plant fitness in natural environments. The rhizosphere, pointed out by Hiltner for the first time, is recognized as a critical region for biogeochemical transformation that underlies the process of soil formation, nutrient cycling, and the ultimate productivity of Earth's terrestrial ecosystems (Hartmann et al., 2008; Shi et al., 2016). The rhizosphere microbiome plays an indispensable role in plant nutrient transformation and absorption, disease control, geochemical cycling of elements, and pollutant degradation (Ezazi et al., 2021; Pang et al., 2021). Therefore, studies have increasingly focused on the effects of agronomic management practices on strong changes in (rhizosphere) soil microbial community structure (Ezazi et al., 2021).

Fertilization is the most common measure used to meet the need for a large number of plants; different fertilization regimes significantly influence crop quality and yield, soil fertility, and microecological health. Excessive application of a single inorganic fertilizer can result in ecological pollution, such as soil acidification, soil compaction, secondary salinization, and degeneration of microbial activity and functional diversity, which impact sustainable soil utilization (Fauci and Dick, 1994; Lupwayi et al., 2011; Gu et al., 2021). Mineral fertilizers consist of simple molecules that are directly available to plants; organic or microbial fertilizers, containing nutrients derived from plants/animal/microbe sources, consist of complex molecules, such as those with a high molecular mass. These compounds serve not only plants but also the soil microbiota as nutrient sources and increase the soil organic matter (SOM) content (Paul Chowdhury et al., 2019). Along with the emergence of novel microbial fertilizers/agents, chemical fertilizer reduction combined with microbial input application has gradually gained much attention and thus become an important assay for improving microecological health and food security in planting production (Chen et al., 2021). For example, Sui et al. (2019) reported that plant growth was notably promoted, and rhizosphere microbial diversity was improved under treatment with the *Bacillus polymyxa* CP-S316 microbial agent. Inoculation of *Sphingomonas* sp. Cra20, a plant growth-promoting rhizobacterium (PGPR), promoted leaf formation, increased the water absorption capacity, and changed the rhizosphere indigenous bacterial community in *Arabidopsis thaliana* (Yang et al., 2019). Different exogenous microbial substances may also have opposite effects on plant growth and nutrient utilization; Weronika et al. (2018) noted that the addition of flavonoids enhanced the flux of amino acids and clover development, while microbial secondary metabolites (phenazine and 2,4-diacetylphloroglucinol) decreased that. Exopolysaccharides fermented by *Pantoea alhagi* NX-11 most increased drought resistance at a 50 mg/L dose; moreover, the total chlorophyll, proline, soluble sugar, antioxidant enzyme, fresh weight, and relative water content all increased (Sun et al., 2020). Welan gum is a kind of microbial exopolysaccharide usually produced by *Alcaligenes* sp. strains. Xu et al. (2020) reported that welan gum could increase the utilization of nitrogen fertilizer and promote rice seedling growth by increasing nitrogen uptake and metabolism, thus representing a promising and ecologically friendly fertilizer. Generally, it has been hypothesized, and a growing body of literature suggests that microbial fertilizer/agent input can improve the soil microenvironment, reduce soil pathogens, and promote proper plant growth and development (Zhao et al., 2014; Chen et al., 2021).

γ-Polyglutamic acid (γ-PGA) is an eco-friendly high-molecular-weight polymer ranging from 10 kDa to 10,000 kDa produced by microbial fermentation. It exhibits extraordinary water and fertilizer retention performance and complete degradability in the environment, thus acting as a novel fertilizer synergist and food preservative in agriculture. γ-PGA is a kind of polypeptide but not a polysaccharid, and it can be excreted extracellularly into the broth supernatant or combined with polysaccharides. γ-PGA application could significantly improve water retention, reduce the drainage rate, prevent soil nutrient loss, and improve crop production and quality (Yin et al., 2018). Xu et al. (2013) concluded that γ-PGA contributed to microbe proliferation, slow release of nutrients, increasing the apparent utilization of nitrogen by 11.38%, and improving wheat field yield by 7.17%. In light of the present situation, studies have mainly focused on the effects of either the pure γ-PGA agent or a compound fertilizer containing γ-PGA on crop production, food security, and soil fertility (Zhang et al., 2017; Yue et al., 2021). The γ-PGA fermentation system, containing both γ-PGA and active functional microorganisms, can be directly applied in agriculture, theoretically overcoming tedious purification operations. If so, what are the efficiency differences between the fermentation agent and its derived compound γ-PGA, and what is the corresponding mechanism? The γ-PGA-producing bacterium *B*. *subtilis* A-5 was isolated from homemade *natto* with high efficiency (>34 g/L) and an ultrahigh molecular weight (4,700 kDa). A previous study showed that purified γ-PGA enhanced the apparent fertilizer utilization rates (N: 27.82–52.27%; P: 17.05–64.59%; K: 32.73–41.43%) and increased the relative abundances of potential plant-growth-promoting taxa in bulk soil in a concentration-dependent manner (Bai et al., 2020). Therefore, a potted microcosmic simulation system was designed; the rhizosphere soil of Chinese cabbage (*Brassica rapa* ssp. *pekinensis*) in different treatments was selected as the research object; the physicochemical properties and enzyme activities were determined; and bacterial community diversity, composition, and correlations were analyzed by high-throughput sequencing. This study will help to understand the mechanism by which γ-PGA and γ-PGA-producing bacteria affect rhizosphere soil metabolism, microbial community structure, and crop fitness, providing a scientific basis for sustainable agricultural development from a rhizosphere microbiota ecology perspective.

## Materials and methods

### Site description

The soil samples were collected from Zhuanghang Comprehensive Experimental Station (30°53'N, 121°23'E). Soil samples were passed through a 2-mm sieve and thoroughly homogenized before establishment of the pot experiments. The soil type is sandy loam. It contained 13.80 g/kg SOM, 0.90 g/kg total nitrogen (TN), 1.60 g/kg total phosphorus (TP), 72.02 mg/kg available nitrogen (AN), 20.74 mg/kg available phosphorus (AP), and 240.00 mg/kg available potassium (AK), with a pH of 7.78 (5:1 water to soil ratio).

### Microbial agent preparation

The γ-PGA-producing strain *B*. *subtilis* A-5 was preserved at the China Center for Type Culture Collection (CCTCC No. 2019157). The bacterium was cultured in LB medium for 48 h at 37 °C and 200 rpm and then centrifuged at 8,000 rpm for 10 min to separate the supernatant, which was discarded; the bacterial liquid was prepared by resuspension using sterilized water and stored at 4°C. The pure γ-PGA product and γ-PGA fermentation broth were prepared according to the methods of a previous study (Bai et al., 2020); the pure γ-PGA product was freeze-dried under vacuum to produce a powder.

### Pot experiment

The experiment was carried out in 67.5 × 17 × 15 cm pots containing 10 kg of soil. Six treatments were established: no fertilization, CK; conventional chemical fertilizer, CF; 30% reduced nitrogen fertilizer, N; 30% reduced nitrogen fertilizer with pure γ-PGA, PGA; 30% reduced nitrogen fertilizer with *B. subtilis* A-5, A5; 30% reduced nitrogen fertilizer with γ-PGA fermentation broth, FJY. The inorganic N, P, and K fertilizers were urea (N% ≥ 46%), calcium superphosphate (P_2_O_5_% ≥ 12%), and potassium chloride (K_2_O% ≥ 60%), respectively. In the CF treatment, N, P_2_O_5_, and K_2_O were applied at 150 mg/kg, 100 mg/kg, and 150 mg/kg, respectively. For the nitrogen reduction treatments, N, P_2_O_5_, and K_2_O were applied at 105 mg/kg, 100 mg/kg, and 150 mg/kg, respectively. Pure γ-PGA, *B*. *subtilis* A-5, and the fermentation broth were applied at approximately 0.60 g, 0.02 L, and 0.01 L, respectively, since the application rate of γ-PGA was 60 mg/kg and the concentration of γ-PGA in the fermentation broth was 30.00–34.21 g/L. Seven seedlings of Chinese cabbage were kept in each pot; the treatment samples were arranged in a randomized design and placed in a greenhouse (25°C, natural light, and 40% relative humidity). Each treatment was replicated three times. Additionally, according to the application rate and the nutrient content in pure γ-PGA and the fermentation broth, these substances were speculated not to interfere with the overall experimental results (data not shown).

### Plant and soil sampling

Chinese cabbage (*Brassica rapa* ssp. *pekinensis*) was harvested 60 days after planting. The rhizosphere soil from each plant was collected into a sterilized container by vigorously shaking the roots. The soil samples were divided into two parts: one portion was stored at −80°C for high-throughput sequencing, and the residues were prepared for soil physicochemical property and enzyme activity determination. Plant samples from each pot were collected separately and washed to remove surface contamination. Thereafter, the yield, leaf area, vitamin C (Vc), and ${\text{NO}}_{3}^{-}$-N indexes were measured by using the fresh weight, a portable leaf area meter, 2,4-dinitrophenylhydrazine, and salicylic acid, respectively. Meanwhile, the TN and TP contents of Chinese cabbage were measured according to the protocols (Gao, 2006). After air-drying and sieving the soil samples with 30-50 mesh, the contents of TN, TP, AN, and AP in the rhizosphere soil were analyzed according to the Analytical Methods of Soil Agricultural Chemistry (Lu, 1999). Sucrase (SC) was measured by the 3,5-dinitrosalicylic acid method as 1 mg of reducing sugar production per gram of soil sample per day. Urease (UE) was determined by the indophenol-blue colorimetry method as 1 μg of ${\text{NH}}_{3}^{+}$-N per gram of soil sample per day.

### Illumina high-throughput sequencing

DNA was extracted from a total of 0.5 g of rhizosphere soil using the MoBio PowerSoil DNA Isolation Kit (MoBio, Carlsbad, CA, USA), according to the manufacturers' protocol. Genomic DNA concentration and quality were measured using a NanoDrop 2000 spectrophotometer (Thermo Scientific, Wilmington, DE, USA). The DNA extracted from each sample was used as a template for amplification of the V3-V4 hypervariable regions of the bacterial 16S rRNA gene. The universal bacterial primers 338F (5'-ACTCCTACGGGAGGCAGC-3') and 806R (5'-GGACTACHVGGGTWTCTAAT-3') were used. The 25-μL reaction system contained 5 μL of 5 × reaction buffer, 2.5 mM dNTPs, 10 μM of each primer (338F/806R), 40 ng of DNA template, and 1 unit of rTaq polymerase (New England Biolabs Co., Ltd., USA). The PCR conditions were as follows: 2 min of initial denaturation at 98°C, followed by 25 cycles of denaturation at 98°C for 15 s, annealing at 55°C for 30 s, and extension at 72°C for 30 s and a final extension at 72°C for 5 min. Paired-end sequencing of bacterial amplicons was carried out using the Illumina MiSeq PE300 platform by Majorbio Bio-Pharm Biotechnology Co., Ltd. (Shanghai, China) according to standard protocols. The raw reads have been deposited into the NCBI Sequence Read Archive (SRA) database (Accession Number: PRJNA842792).

### Bioinformatics analysis

After demultiplexing, the resultant sequences were assembled using FLASH v1.2.11 (Magoc and Salzberg, 2011) and quality filtered with fastp (0.19.6) (Chen et al., 2018). Then, the high-quality sequences were denoised using the DADA2 (Callahan et al., 2016) plugin in the QIIME2 (v2020.2) (Bolyen et al., 2019) pipeline with the recommended parameters, which yielded single-nucleotide-resolution data based on error profiles within samples. DADA2-denoised sequences are usually called amplicon sequence variants (ASVs). Taxonomic assignment of ASVs was performed using the naive Bayes consensus taxonomy classifier implemented in QIIME2 and the SILVA 16S rRNA database (v138), and the classification confidence was 0.7.

### Statistical analysis

The richness, diversity, and evenness of the bacterial community were estimated using the Chao1, Shannon, Simpson, and Simpsoneven indexes, which were calculated based on the rarefied ASVs. Principal coordinate analysis (PCoA) based on the Bray–Curtis matrix was performed to determine the β diversity. Permutational multivariate analysis of variance (PERMANOVA) was also used to detect differences in bacterial community structure in the rhizosphere soil under different treatments (Ramette, 2007). The relationships between environmental factors, dominant taxa, and bacterial community distribution in the rhizosphere soil were investigated using the variance inflation factor (VIF), redundancy analysis (RDA), and Pearson correlation analysis approaches. Finally, the FAPROTAX database was used to predict the functions of rhizosphere soil microbes.

Statistically significant differences were analyzed using SPSS 19.0 software (SPSS Inc., Chicago, IL, USA) with one-way ANOVA by the Tukey HSD test. Graphic layouts were generated using Adobe Illustrator CS6 (Adobe Systems Incorporated, USA). The online Majorbio Cloud Platform (cloud.majorbio.com, 17 August 2021) was used for bioinformatic analyses (bar plot, PCoA, RDA, and FAPROTAX diagrams).

## Results

### Chinese cabbage growth characteristics

The aboveground parts of plants positively respond to belowground plant–microbe interactions in the rhizosphere. Treatments affected the yield and quality of Chinese cabbage differently (Table 1). Compared to the N treatment, microbial agent (PGA, A5, and FJY) addition significantly increased the crop yield (by 20.19–37.63%), which was comparable to or higher than the effects of the CF treatment (*P* < 0.05). The FJY treatment had the highest vegetable yield (26385.09 kg/hm^2^) among the six treatments, which was notably higher than the yield in the PGA and A5 treatments by 9.00–12.67% (*P* < 0.05). There was no significant difference between the A5 and PGA treatments, suggesting similar growth-promoting effects.

**Table 1:** Effects of different treatments on the yield and quality of Chinese cabbage.

**Treatment**	**Yield kg·hm^–2^**	**Leaf area cm^2^**	**${\text{NO}}_{\text{3}}^{-}$-N mg·kg^–1^**	**Vc μg·g^–1^**
CK	16106.73 ± 330.83d	40.87 ± 1.62e	50.11 ± 11.08d	342.53 ± 36.53a
CF	22923.10 ± 503.00b	53.30 ± 1.94d	439.03 ± 20.74a	329.61 ± 29.93a
N	19171.35 ± 1278.49c	52.40 ± 2.30d	69.59 ± 14.92d	367.83 ± 27.23a
PGA	23042.98 ± 1498.00b	64.45 ± 2.85c	131.67 ± 5.62bc	356.68 ± 28.70a
A5	24009.36 ± 1182.21b	105.17 ± 2.87a	144.80 ± 8.75b	372.17 ± 22.33a
FJY	26385.09 ± 920.21a	94.22 ± 2.90b	95.65 ± 13.35cd	377.14 ± 21.54a

No fertilization, CK; conventional chemical fertilizer, CF; 30% reduced nitrogen fertilizer, N; 30% reduced nitrogen fertilizer with pure γ-PGA, PGA; 30% reduced nitrogen fertilizer with Bacillus subtilis A-5, A5; 30% reduced nitrogen fertilizer with γ-PGA fermentation broth, FJY. The data are the mean ± SD; lowercase letters indicate statistically significant differences among treatments at the 0.05 level.

Biological agent application and fertilization significantly increased the leaf area in the order A5>FJY>PGA>CF/N>CK. The leaf area in the A5 treatment was the highest (105.17 cm^2^), which was 1.12–2.57 times those in the other treatments. Green vegetables more easily enrich nitrate than other crops, so the nitrate content has become an important indicator of vegetable quality. As shown in Table 1, the nitrate content in the CF treatment was the highest (439.03 mg/kg, *P* < 0.05), followed by that in the A5 treatment (144.80 mg/kg). Nitrate accumulation in vegetables can be affected by the nitrogen application level; a positive correlation between the nitrate content in green vegetables and the nitrogen application rate was observed in this study, which was consistent with the conclusions of previous research (Kuscu et al., 2014). Nitrogen reduction significantly decreased the nitrate content in Chinese cabbage, as shown in the N, PGA, A5, and FJY treatments, ranging from 69.59 mg/kg to 144.80 mg/kg (*P* < 0.05). Although the application of biological agents (PGA, A5, and FJY) increased the nitrate level compared with N treatment, the level was still notably lower than that in the CF treatment (*P* < 0.05). The application of active bacteria (A5 and FJY treatments) was slightly beneficial for increasing the Vc content, but there were no significant differences among the six treatments (Table 1).

### Physicochemical properties and enzyme activity of the rhizosphere soil

Biological agent addition resulted in significant variation in the pH value of the rhizosphere soil, with the PGA treatment increasing the rhizosphere soil pH but the active bacterial treatments (A5 and FJY) decreasing it (*P* < 0.05). The variation in TN, TP, AN, and AP contents showed a tendency similar to that of pH (Table 2). Specifically, compared with the N treatment, the PGA treatment significantly increased the AP content by 50.03%, and the FJY treatment significantly decreased the TN and AN contents (0.87 mg/kg, 40.21 mg/kg) and increased the AP content (95.76 mg/kg). Compared to CF, the N treatment significantly decreased the SOM content by 10.05% and increased the AP content by 46.47% (*P* < 0.05).

**Table 2:** Effects of different treatments on rhizosphere soil physicochemical properties.

**Treatment**	**pH**	**SOM %**	**TN g·kg^–1^**	**TP g·kg^–1^**	**AN mg·kg^–1^**	**AP mg·kg^–1^**
CK	8.28 ± 0.01c	5.33 ± 0.31b	0.84 ± 0.04c	2.61 ± 0.05c	36.45 ± 3.47c	33.85 ± 4.52c
CF	8.37 ± 0.03ab	5.97 ± 0.21a	1.03 ± 0.11ab	2.82 ± 0.06ab	45.60 ± 7.35ab	46.22 ± 15.76c
N	8.35 ± 0.02b	5.37 ± 0.15b	1.01 ± 0.07ab	2.79 ± 0.02ab	50.81 ± 5.96a	67.72 ± 6.64b
PGA	8.40 ± 0.04a	5.60 ± 0.35ab	1.05 ± 0.04a	2.86 ± 0.06a	53.02 ± 3.68a	101.60 ± 10.84a
A5	8.27 ± 0.01c	5.63 ± 0.25ab	0.94 ± 0.06bc	2.66 ± 0.06bc	47.20 ± 1.77ab	80.05 ± 4.22b
FJY	8.30 ± 0.01c	5.70 ± 0.20ab	0.87 ± 0.02c	2.72 ± 0.08b	40.21 ± 1.72bc	95.76 ± 1.24a

SOM, TN, TP, AN, and AP refer to soil organic matter, total nitrogen, total phosphorus, available nitrogen, and available phosphorus, respectively. The data are the mean ± SD; lowercase letters indicate statistically significant differences among treatments at the 0.05 level.

**Figure 1:**
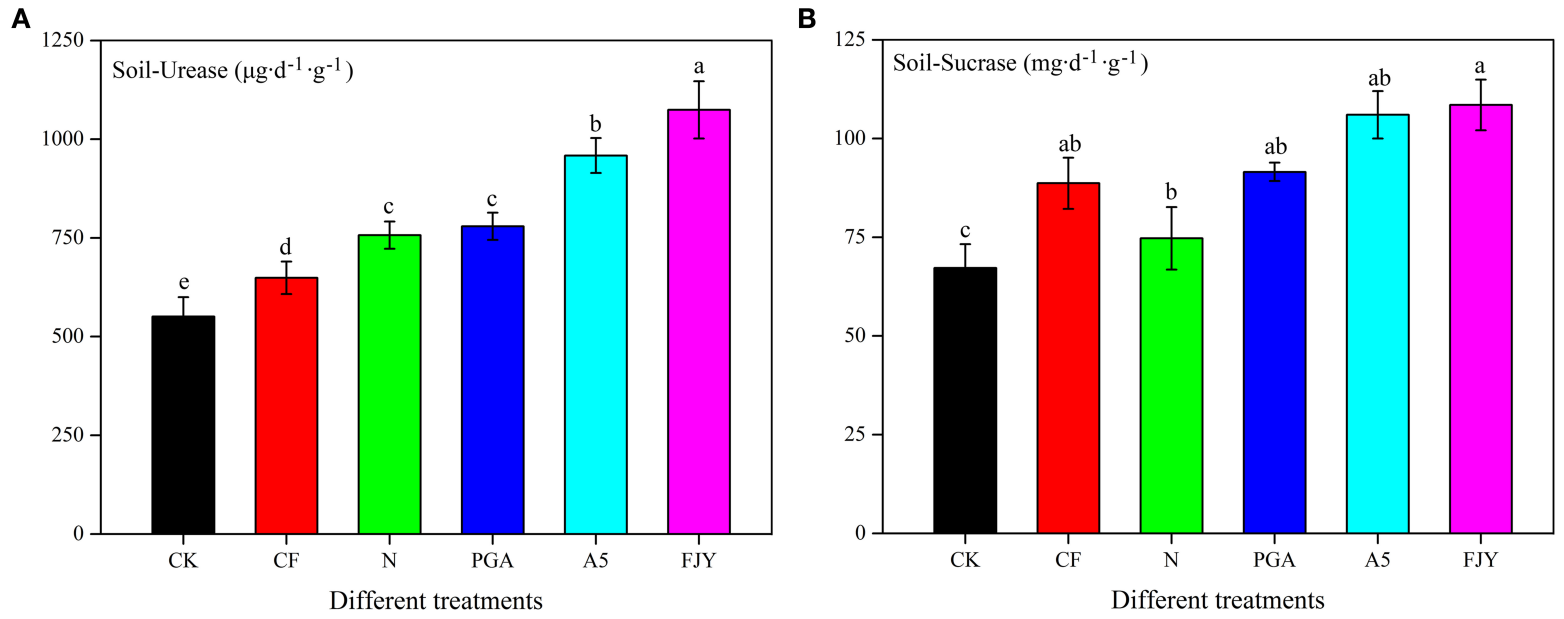
Effects of different treatments on urease activity **(A)** and sucrase **(B)** in the rhizosphere soil.

UE can hydrolyze urea to ammonia in soil, thus reflecting the soil nitrogen status. As shown in Figure 1, the UE activity was lowest (550.82 μg/d·g) under the no fertilization treatment (CK), and fertilization practices significantly increased the UE activity (*P* < 0.05). Compared to the N treatment, the A5 and FJY treatments significantly enhanced UE activity by 26.65% and 41.93%, respectively (*P* < 0.05), while the PGA treatment exhibited no significant influence. SC, an important index for evaluating soil fertility, is reported to be related to the SOM, N, and P contents, microorganism number, and respiration intensity of soil. SC plays an important role in increasing soluble nutrients in soil. Similarly, the fertilizer application dose increased SC activity compared to that in CK; the FJY treatment markedly improved the SC activity by 45.22% in comparison with that of the N treatment (*P* < 0.05) (Figure 1).

### α-diversity analysis of the bacterial community in the rhizosphere soil

**Figure 2:**
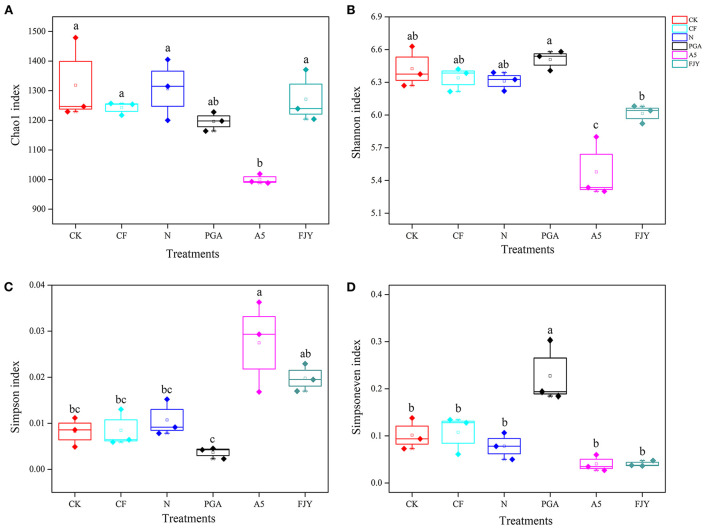
Comparison of bacterial community ? diversity Chao1 index **(A)**, Shannon index **(B)**, Simpson index **(C)**, and Simpsoneven index **(D)**, in Chinese cabbage rhizosphere soil.

Prokaryotes contribute to a large portion of the enormous biodiversity observed in soils and are involved in various important ecosystem functions, such as nutrient cycling and plant health. After quality filtering, a total of 484,176,900 bp of high-quality sequences were obtained from the 18 rhizosphere soil samples. After rarefaction, each sample contained 28,839 sequences. The high-quality sequences across all samples were identified to have an average of 5834 ASVs. The α-diversity values of the rhizosphere soil bacterial community between treatments were statistically analyzed and are detailed in Figure 2. The Chao1 index indicates microbial community richness; nitrogen reduction showed no effects on the bacterial community richness compared with the CF treatment, and the A5 treatment significantly reduced the Chao1 index to 1000.10 (*P* < 0.05), while the reduction in the PGA treatment was insignificant (8.44%) compared with the N treatment. The Shannon and Simpson indexes can reflect microbial community diversity. Specifically, the higher the Shannon index is, the higher the diversity, and the higher the Simpson index is, the lower the diversity. Compared with N treatment, the PGA treatment had no significant effects on rhizosphere bacterial diversity, while the A5 and FJY treatments decreased the Shannon index by 4.75–13.15% and increased the Simpson index by 81.82–145.45%, with the A5 treatment being notably different (*P* < 0.05). The PGA treatment significantly increased the bacterial community evenness (Simpsoneven index) (*P* < 0.05). In the A5 and FJY treatments, the variation in the Simpsoneven index was not statistically significant (Figure 2D).

### Analysis of bacterial community structure in the rhizosphere soil

**Figure 3:**
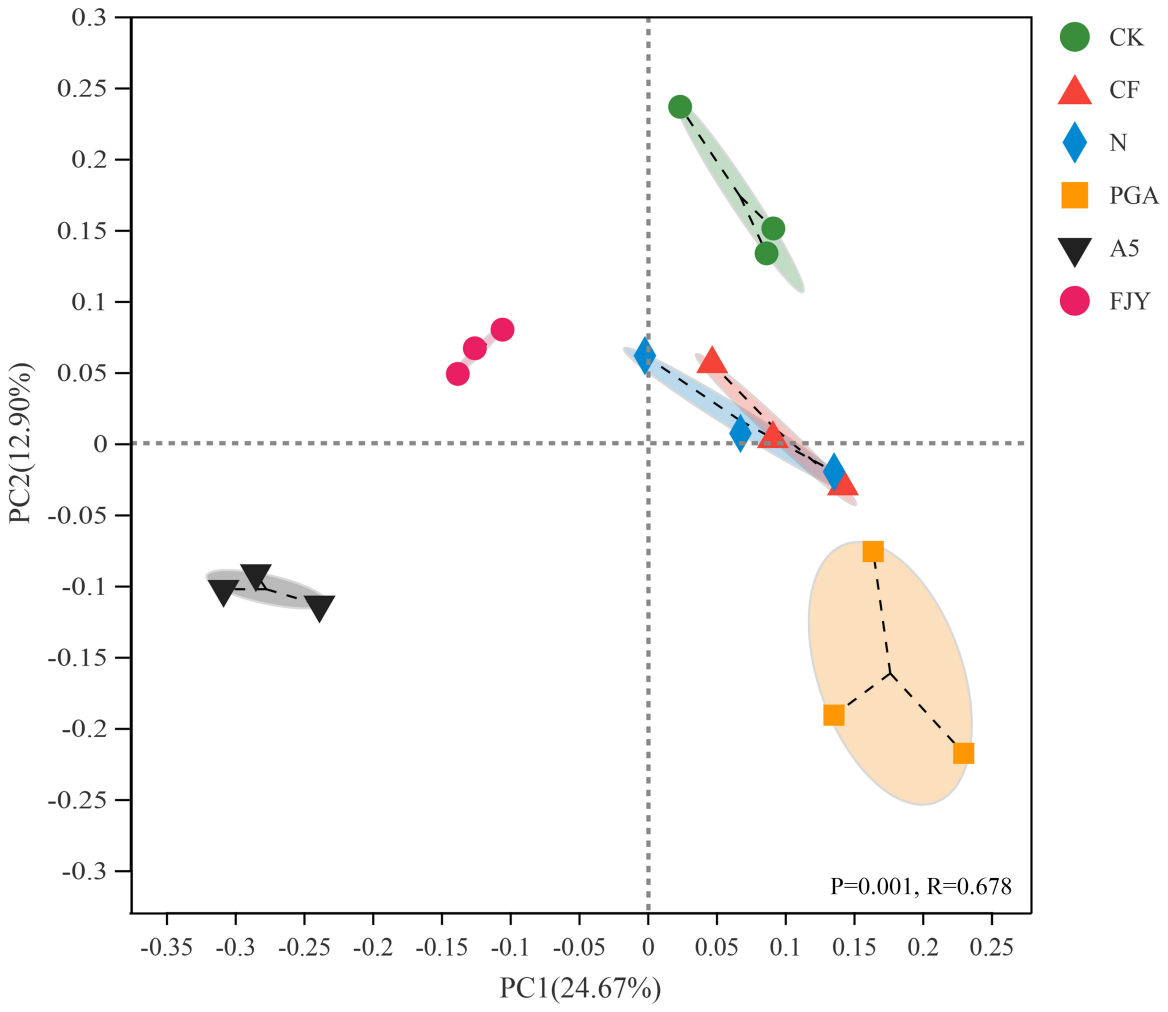
PCoA of bacterial community structure in the rhizosphere soil based on the Bray–Curtis matrix.

As shown in Figure 3, PCoA with the Bray–Curtis algorithm demonstrated that the bacterial communities from different treatments were clearly separated, except those under the CF and N treatments. The first two axes, PC1 and PC2, explained 24.67% and 12.90% of the total variation in the bacterial community structure, respectively. PC1 distinguished the microbial agent treatments (A5 and FJY) from the other four treatments (CK, CF, N, and PGA), suggesting that exogenous functional bacteria reshaped the original indigenous microbiome in the rhizosphere soil. PC2 differentiated A5 and PGA treatments from FJY and CK treatments. The similar trends in bacterial community structure in the CF and N treatments indicated that rhizosphere microbes of Chinese cabbage were probably insensitive to the nitrogen application rate in this study and that the rhizospheric bacterial community was relatively ecologically stable. PERMANOVA again proved the significant differences in bacterial community structure among the different treatments (*P* = 0. 001, *R* = 0.678).

### Analysis of bacterial community composition in the rhizosphere soil

**Figure 4:**
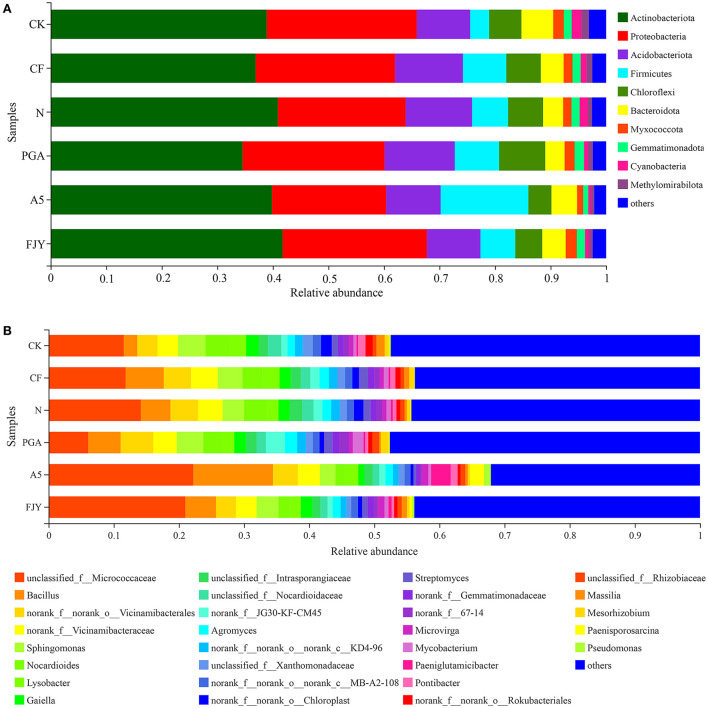
The average relative abundances of bacteria at the phylum **(A)** and genus **(B)** levels for rhizosphere soil samples under different treatments. “Others” includes phyla/genera with a relative abundance below 1%.

Microorganisms in the plant rhizosphere directly and effectively transform soil nutrients and may also promote nutrient absorption by releasing exudates to induce the “rhizosphere effect”, thus accelerating proper plant development. The relative abundances of bacterial community composition at the phylum level were analyzed and are displayed in Figure 4A; taxa with an abundance of <1% merged into “others”. Bacterial sequences across all rhizosphere soils were classified into 32 phyla, with the most dominant phyla Actinobacteria and Proteobacteria occupying 34.55–40.90% and 20.55–27.03%, respectively. Treatments did not affect the phylum-level bacterial community composition but obviously affected the relative abundances of different phyla. One-way ANOVA of the top 10 phyla taxa showed that the relative abundances of Chloroflexi, Myxococcota, Cyanobacteria, Methylomirabilota, and Gemmatimonadota were significantly influenced by fertilization regime, especially microbial agent application (*P* < 0.05) (Supplementary Table 1). Nitrogen reduction appears to have no obvious effect on the rhizospheric bacterial taxa, as shown in the comparison between the N and CF treatments. Compared with the N treatment, A5 treatment increased and decreased the relative abundances of Firmicutes and Gemmatimonadota to 12.81% and 0.94%, respectively. Regarding the differences between microbial agent treatments, PGA treatment exhibited a higher content of Chloroflexi than A5 and FJY treatments (*P* < 0.05).

The relative abundances of the dominant genera were analyzed and are presented in Figure 4B, with genera showing an abundance less than 1% merged into “others”. At the genus level, although the bacterial community composition was similar among the different groups, the distribution of each genus differed by soil sample. The genera with relative abundances >2% were unclassified_f_Micrococcaceae (14.47%), *Bacillus* (5.73%), norank_f_norank_o_Vicinamibaceales (3.90%), norank-f_Vicinamibaceae (3.53%), *Sphingomonas* (3.53%), *Nocardioides* (2.73%), and *Lysobacter* (2.08%). Statistical analysis showed that the relative abundances of unclassified_f_Micrococcaceae, *Bacillus, Nocardioides, Gaiella*, and unclassified_f__Nocardioidaceae were significantly different among the six treatments (Supplementary Table 2). In comparison with the N treatment, PGA and FJY treatments exhibited certain influences but not to a significant degree, and A5 treatment obviously enhanced the *Bacillus* content and reduced the relative abundances of *Nocardioides* and *Gaiella* (*P* < 0.05). In addition, PGA and A5 treatments also significantly affected some bacterial orders, such as Micrococcales, Propionibacteriales, Burkholderiales, Gaiellales, and Microtrichales (Supplementary Figure 1).

### Correlation analysis of environmental factors and rhizosphere soil community structure

**Figure 5:**
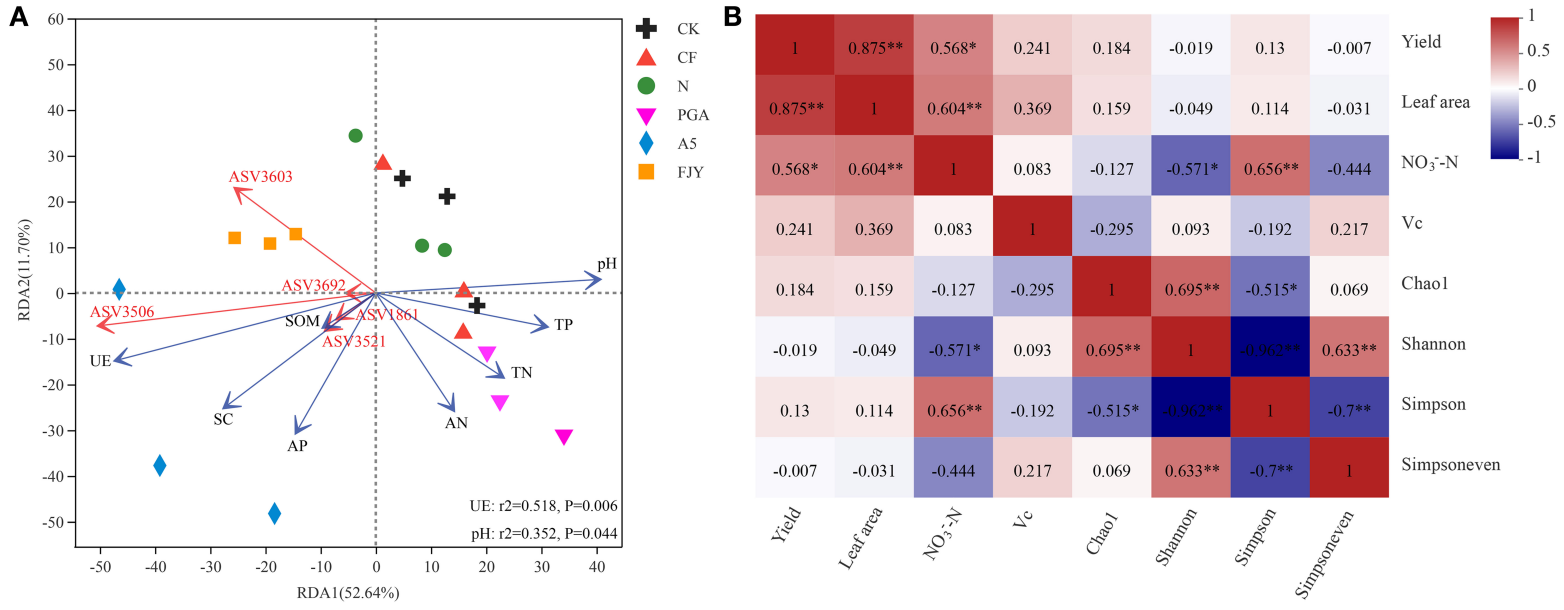
RDA of rhizosphere soil elements and bacterial community under different fertilization treatments **(A)**; Pearson correlation analysis between vegetable yield and quality and bacterial community α diversity in the rhizosphere soil **(B)**. “*” and “**” indicate significant differences (*P* < 0.05) and extremely significant differences (*P* < 0.01), respectively.

The effects of environmental factors on bacterial community structure in the rhizosphere soil under different fertilization treatments were studied, followed by VIF analysis to eliminate the factors with strong collinear relationships. Figure 5A presents the RDA results for the relationships between the environmental factors and rhizosphere soil microbes at the ASV level. RDA1 and RDA2 explained 52.64% and 11.70% of the variation in bacterial community structure, respectively, with a cumulative interpretation percentage of 64.34%. Strain A-5 addition treatments (A5 and FJY) were separated by RDA1 from the other treatments, indicating distinct differences in bacterial community structure. Furthermore, environmental factors such as pH, SOM, TN, TP, AN, AP, UE, and SC impacted bacterial community structure and species distribution, with UE (*r*^2^ = 0.518, *P* = 0.006) and pH (*r*^2^ = 0.352, *P* = 0.044) exhibiting the greatest influence. Rhizosphere soil properties, except pH, were negatively correlated with RDA2, among which available nutrients (AP and AN) had the highest negative correlation with RDA2. pH, TP, TN, and AN were positively correlated with RDA1, while UE, SC, SOM, and AP showed negative correlations. Moreover, the top 5 ASVs (ASV3603, 3506, and 3692 belonging to unclassified_f__Micrococcaceae; ASV3521 belonging to *Paeniglutamicibacter*; ASV1861 belonging to *Bacillus*) seemed to be enriched in the microbial agent treatments (A5 and FJY) rather than the other four treatments.

Furthermore, a Pearson correlation analysis was used to determine the relationships between Chinese cabbage production parameters and the α diversity of the rhizospheric bacterial community (Figure 5B). The vegetable yield was not related to any of the bacterial community α diversity indexes (Chao1, Shannon, Simpson, and Simpsoneven), but was positively correlated with leaf area (*P* < 0.01) and ${\text{NO}}_{3}^{-}$-N content (*P* < 0.05). ${\text{NO}}_{3}^{-}$-N was notably correlated with bacterial community diversity (Shannon and Simpson indexes), with ${\text{NO}}_{3}^{-}$-N and the Simpson index showing an extremely significant positive correlation (*r* = 0.656, *P* < 0.01). The Vc content presented a distinct correlation with neither vegetable production nor the α diversity of the rhizosphere bacterial community.

### FAPROTAX function prediction

**Figure 6:**
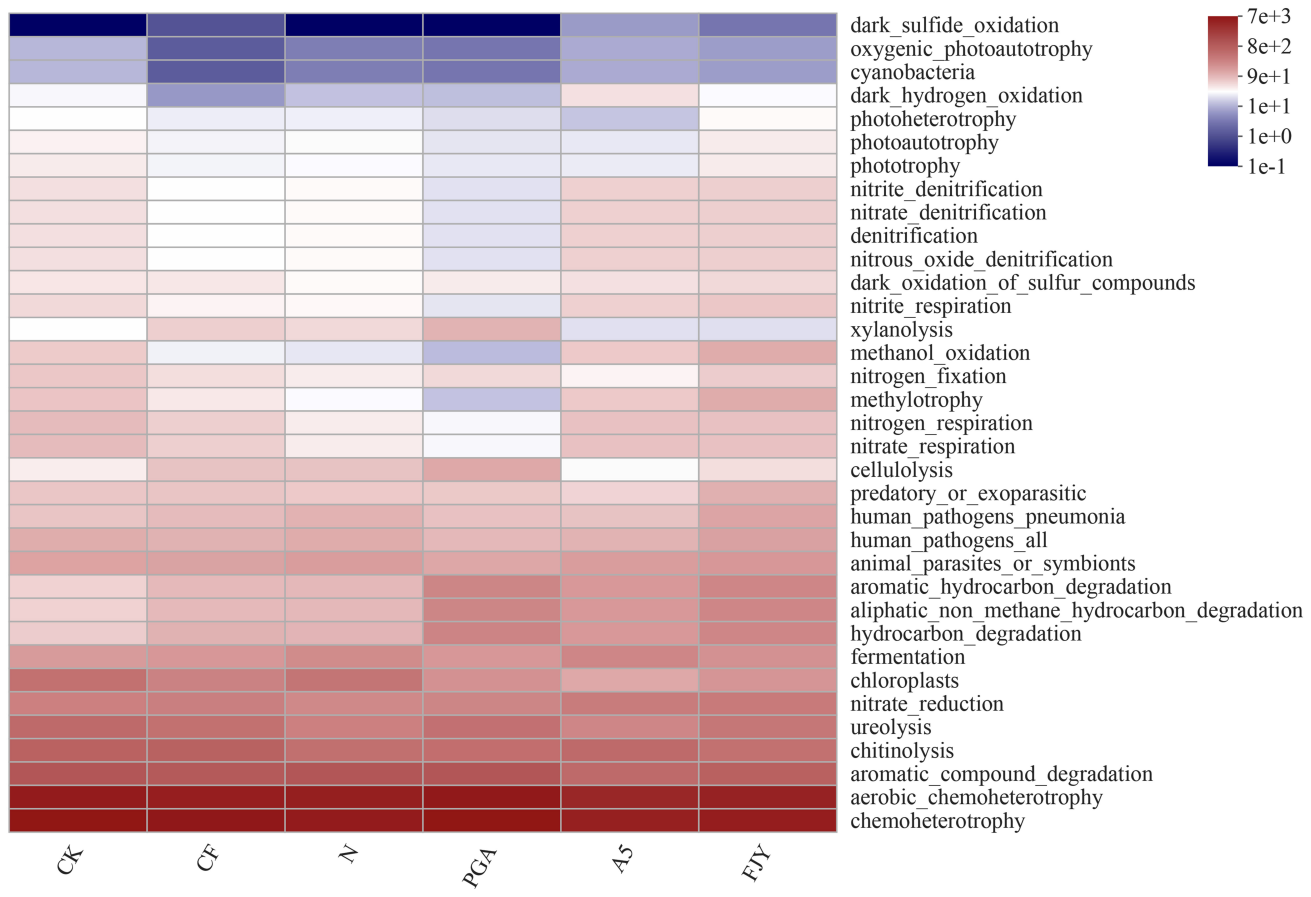
Functional heatmap of the rhizosphere soil predicted by the FAPROTAX database under different treatments.

The FAPROTAX database is used to map prokaryotic genera or species to metabolism or other ecologically relevant functions, such as nitrification and denitrification (Sansupa et al., 2021). The functions of bacteria in Chinese cabbage rhizosphere soil with different treatments were annotated by FAPROTAX, and the top 20 functions in terms of relative abundance were analyzed statistically (Supplementary Figure 2). In Figure 6, the dominant bacterial functions were mainly related to chemotrophy and heterotrophy, especially material decomposition (aromatic substances, hydrocarbons, chitin, and cellulose) and nitrogen cycling (urea decomposition, nitrate reduction, nitrate respiration, and nitrogen respiration), with significant or extremely significant differences driven by fertilization and microbial agent application (*P* < 0.05; *P* < 0.01). Furthermore, the expression of genes that function in N cycling [archaeal and bacterial *amoA* (AOA + AOB), *nifH*, and *nosZ*] was influenced by fertilization and microbial agents; in particular, *amoA* (AOB) exhibited a significant increase compared with that of the CK treatment (Supplementary Figure 3).

## Discussion

Microbial composition and diversity in the rhizosphere zone are important indicators of soil ecological function and are strongly regulated by biotic and abiotic factors (Hamonts et al., 2018). Interactions between roots, microorganisms, and the soil matrix represent a suite of extremely complex processes (Shi et al., 2016). Therefore, the analysis of the rhizosphere microbial community has increasingly become an important technical method for evaluating the functions of novel agents/fertilizers. Single (reduction) fertilization did not significantly affect the α diversity of the rhizosphere soil bacterial community compared to that in the CK treatment; fertilization and microbial agents had a combinatorial effect on bacterial richness, diversity, and evenness. Generally, FJY treatment slightly affected the bacterial community diversity; A5 treatment tended to decrease the bacterial community richness (Chao1) and diversity (Shannon and Simpson); PGA treatment affected the bacterial evenness more sensitively than A5 and FJY treatments did (*P* < 0.05). The addition of exogenous microorganisms (*B*. *subtilis* A-5) may significantly affect the community structure of rhizosphere microorganisms because of their need to compete with indigenous microbes to colonize and survive in the rhizosphere (Wu et al., 2021). For example, Li, 2021 found that in Ningnan tobacco fields, *B*. *amyloliticus* B1619 application decreased bacterial community α diversity during the flourishing period, while compound biological agents increased the α diversity of the rhizosphere bacterial community during the clumping and flourishing periods, indicating that specific microbial agents may differ in their effects on the bacterial community of rhizosphere soil. The quantity and category of the rhizospheric microflora were preferentially selected according to crop species and growth periods (Yuan et al., 2015), as well as the agent types (Yin et al., 2018; Bai et al., 2020). Therefore, planting crops, inoculation time, and developmental stage are all important factors and should be considered in further analyses.

The balance between beneficial and deleterious rhizosphere microorganisms is critical for healthy crops. Fertilization and biological agents in combination affected the bacterial community composition (Figure 4). Most species in Chloroflexi are unculturable and widely exist in sewage treatment plants, rivers, soil, deep seas, and hot springs, among other habitats. Furthermore, they are dominant in reclaimed farmland soils, where they may be related to various biotechnological processes, such as nutrient metabolism, biogeochemical chlorine cycling, or degradation of soluble microbial products (Trivedi et al., 2016). The presence of *B*. *subtilis* A-5 (A5 and FJY treatments) tended to decrease the relative abundance of Chloroflexi; PGA treatment increased it but was not significantly different from that in the N treatment (6.28%). Some genera in Myxococcota, such as *Myxobacteria*, exhibit predation and soil adaptability, indicating their application potential in microbial ecological network regulation and plant disease control (Livingstone et al., 2017). Compared with that in CK (1.90%), the relative abundance of Myxococcota in the A5 treatment significantly decreased to 1.09%. However, as shown in Figure 6 and Supplementary Figure 2, the relative abundance of the predatory or exoparasitic function was highest in the FJY treatment, which is consistent with the highest relative abundance of Myxococcota (1.98%) in this treatment. Thus, FJY treatment may be more conducive to the optimization and stability of the rhizosphere soil microbial ecological network. Methylomirabilota can anaerobically oxidize methane coupled with nitrate/nitrite reduction, so its abundance influences nitrogen and carbon cycling driven by microorganisms (Ivanova et al., 2022). Compared with CK, the fertilization treatments all reduced the relative abundance of Methylomirabilota, especially the nitrogen reduction and exogenous biological agent treatments (N, PGA, A5, and FJY), which showed evident differences (0.55-0.84%). The relatively low content of Methylomirabilota in A5 and FJY treatments suggested an influence of *B*. *subtilis* A-5 on nitrogen and carbon cycling. Fertilizer application also affected bacterial community composition at the genus level in the rhizosphere soil. Micrococcaceae is a common PGPR; in the present study, it was enriched and reduced by *B*. *subtilis* A-5 and pure γ-PGA, respectively. Furthermore, in Figure 5A, ASV3521 belonged to *Paeniglutamicibacter*, which is a genus of high G+C gram-positive bacteria in the family Micrococcaceae. Therefore, strain A-5 and Micrococcaceae might synergistically promote survival in Chinese cabbage rhizospheric soil. The relative abundance of Nocardioides showed a downward trend in A5 and FJY treatments; Nocardioides belongs to the Nocardioidaceae family, which is typically related to nitrogen cycling. γ-PGA and *B*. *subtilis* A-5 application dramatically affected functions related to carbon sequestration and nutrient cycling in the rhizosphere bacterial community, such as ureolysis, nitrate reduction, nitrate respiration/nitrogen respiration (Figure 6) and N cycling genes (Supplementary Figure 3). Although the relative abundance of *Bacillus* was significantly increased in A5 treatment, the dynamic variation in *B*. *subtilis* A-5 was not analyzed in detail in this study. Subsequently, the colonization and growth promotion mechanisms of *B*. *subtilis* A-5 need to be explored by fluorescent protein labeling or metagenomic analysis.

Furthermore, the application of functional microbial agents can result in altered growth, functional traits and nutritional quality of aboveground plant parts (Lu et al., 2018). Compared with N treatment, the presence of *B*. *subtilis* A-5 (A5 and FJY treatments) decreased the TN and TP contents in rhizosphere soil (Table 2). In contrast, some studies reported that the addition of exogenous functional bacteria increases the nitrogen and phosphorus contents in rhizosphere soil (Li, 2021), which may be related to different sampling periods; overall, the addition of exogenous biological agents significantly increased the yield of Chinese cabbage (Table 1). Nitrogen reduction and microbial agent addition had notable influences on the AP content in the rhizosphere soil. The N treatment increased the AP content by 46.52% compared to that in CF (*P* < 0.05); all of the microbial agent treatments enhanced the AP content, in comparison with N treatment, with PGA and FJY treatments showing significant differences (41.41–50.03%) (*P* < 0.05). This phenomenon might be related to the capability of high-molecular-weight γ-PGA to complex elements and prevent leaching. In our previous work, we noted that the γ-PGA produced by strain A-5 facilitated vegetable resistance to high temperature compared with the commercial γ-PGA products (data not shown). The application of microbial fertilizer would improve the nutrients needed for microbial reproduction, promote microbial metabolism, reshape the rhizosphere microbiome, and thus strengthen soil enzyme activities (Yang et al., 2019).

UE activity and pH were the key environmental factors affecting the bacterial community structure in rhizosphere soil (Figure 5A). Soil pH is a common and important factor affecting the structure and distribution of the bacterial community (Xun et al., 2015). Wang et al. (2019) emphasized that soil pH was more important than nutrients in shaping bacterial communities in agricultural soils, including their ecological functions and biogeographic distribution. UE, which is widely distributed in soil, degrades urea and is considered to be a good proxy of N mineralization. The importance of UE activity was also evidenced by the variation in nitrogen cycling genes in the FAPROTAX functional prediction analysis, as depicted in Figure 6. Nevertheless, Xun et al. (2015) concluded that the composition and function of soil microorganisms mainly depended on soil intrinsic properties rather than the microbial inoculum. However, that study was carried out with bulk soil in the laboratory and omitted the induction of root exudation and directional assembly of the rhizosphere flora. The rhizosphere microbiome is distinct from microbial communities found in the rest of the soil and is even more important for plant nutrient uptake and health (Shi et al., 2016). γ-PGA can facilitate the formation of soil aggregates to conserve soil fertility, promote plant fitness, and modulate microbial community structure (Yue et al., 2021). *B*. *subtilis* was recognized to promote plant growth or biocontrol of soil-borne disease (Balderas-Ruíz et al., 2021). In our previous research, strain A-5 was also used as a multifunctional PGPR with biocontrol properties and salt-alkaline resistance (Bai et al., 2022). Therefore, A-5 addition (A5 and FJY treatments) significantly altered the bacterial community structure in the rhizosphere soil. Root exudates were also very important for the microbes accumulated in the rhizosphere. The combination of plant exudation traits and microbial substrate uptake traits interacted to yield the patterns of microbial community structure (Zhalnina et al., 2018). However, soil metabolite variation was not examined in this study. In the interaction between strain A-5 and plant roots, the synergistic mechanism between strain A-5 and γ-PGA should be further studied using, for example, stable isotopes, comparative omics, and fluorescent protein tracking methods.

## Conclusion

The PGA, A5, and FJY treatments all significantly promoted crop growth, and the FJY treatment showed the best growth-promoting effect, resulting in a Chinese cabbage yield of 26385.09 kg·hm^−2^ (*P* < 0.05). FJY treatment notably improved soil fertility, increased soil enzyme activity, and improved the quality parameters of Chinese cabbage. Compared with N treatment, A5 treatment decreased rhizosphere bacterial community richness and diversity to a notable degree; PGA treatments increased bacterial evenness; FJY showed no significant effects on bacterial community α diversity. PCoA showed that the addition of *B*. *subtilis* A-5 (A5 and FJY treatments) significantly affected the community structure. At the phylum level, the abundances of Actinobacteria, Proteobacteria, Acidobacteria, Firmicutes, and Chloroflexi were the dominant taxa; microbial agents changed the relative abundances of unclassified_f_Micrococcaceae, *Nocardioides, Bacillus, Gaiella*, and unclassified_f__Nocardioidaceae, thus reshaping and balancing the rhizosphere soil microorganisms and affecting nutrient transformation and cycling. Urease activity and soil pH were the key factors affecting rhizosphere soil bacterial community structure. In conclusion, γ-PGA fermentation broth containing both γ-PGA and active functional bacteria, which eliminates the need for purification operations, represents a potential candidate green fertilizer for future use.

## Data availability statement

The datasets presented in this study can be found in online repositories. The names of the repository/repositories and accession number(s) can be found in the article/Supplementary material.

## Author contributions

NB and HanZ contributed to analysis, interpretation of data, and writing original draft of the manuscript. WL and SL contributed to the conception and design of the study. YH, JZ, and XZ contributed to field investigation and sample acquisition. HaiZ and YZ contributed to the editing and revising of the manuscript. All authors contributed to the article and approved the submitted version.

## Funding

This research was supported by Scientific and Technological Innovation Community Project of Yangtze River Delta of Shanghai Science and Technology Commission (21002410300), Shanghai Agriculture Applied Technology Development Program, China (Hu-Nong-Ke-Tui 2021-2-2), and the Outstanding Team Program of Shanghai Academy of Agricultural Sciences (Hu-Nong-Ke-Zhuo 2022-008). The funding sources had no role in the study design, data collection, analysis, and interpretation, or the decision to submit the article for publication.

## Conflict of interest

The authors declare that the research was conducted in the absence of any commercial or financial relationships that could be construed as a potential conflict of interest.

## Publisher's note

All claims expressed in this article are solely those of the authors and do not necessarily represent those of their affiliated organizations, or those of the publisher, the editors and the reviewers. Any product that may be evaluated in this article, or claim that may be made by its manufacturer, is not guaranteed or endorsed by the publisher.

## Supplementary material

Supplementary Figure 1 One-way ANOVA of the top 10 bacterial taxa with significant differences at the order level. “^*^” and “^**^” indicate significant differences (*P* < 0.05) and extremely significant differences (*P* < 0.01), respectively.

Supplementary Figure 2 Statistically significant difference analysis in rhizosphere soil bacterial functions under different treatments. “^*^” and “^**^” indicate significant differences (*P* < 0.05) and extremely significant differences (*P* < 0.01), respectively.

Supplementary Figure 3 qPCR analysis of the expression of N cycling functional genes (*amoA* [AOA, AOB], *nifH*, and *nosZ*).

Supplementary Table 1 One-way ANOVA of the top 10 bacterial taxa with significant differences at the phylum level (%).

Supplementary Table 2 One-way ANOVA of the top 10 bacterial taxa with significant differences at the genus level (%).
