# Supplementary figures and images for: Effects of Bacillus subtilis A-5 and its fermented γ-polyglutamic acid on the rhizosphere bacterial community of Chinese cabbage
Source: Front Microbiol. 2022 Aug 15;13:954489. doi: 10.3389/fmicb.2022.954489 (PMC9421268; doi:10.3389/fmicb.2022.954489)

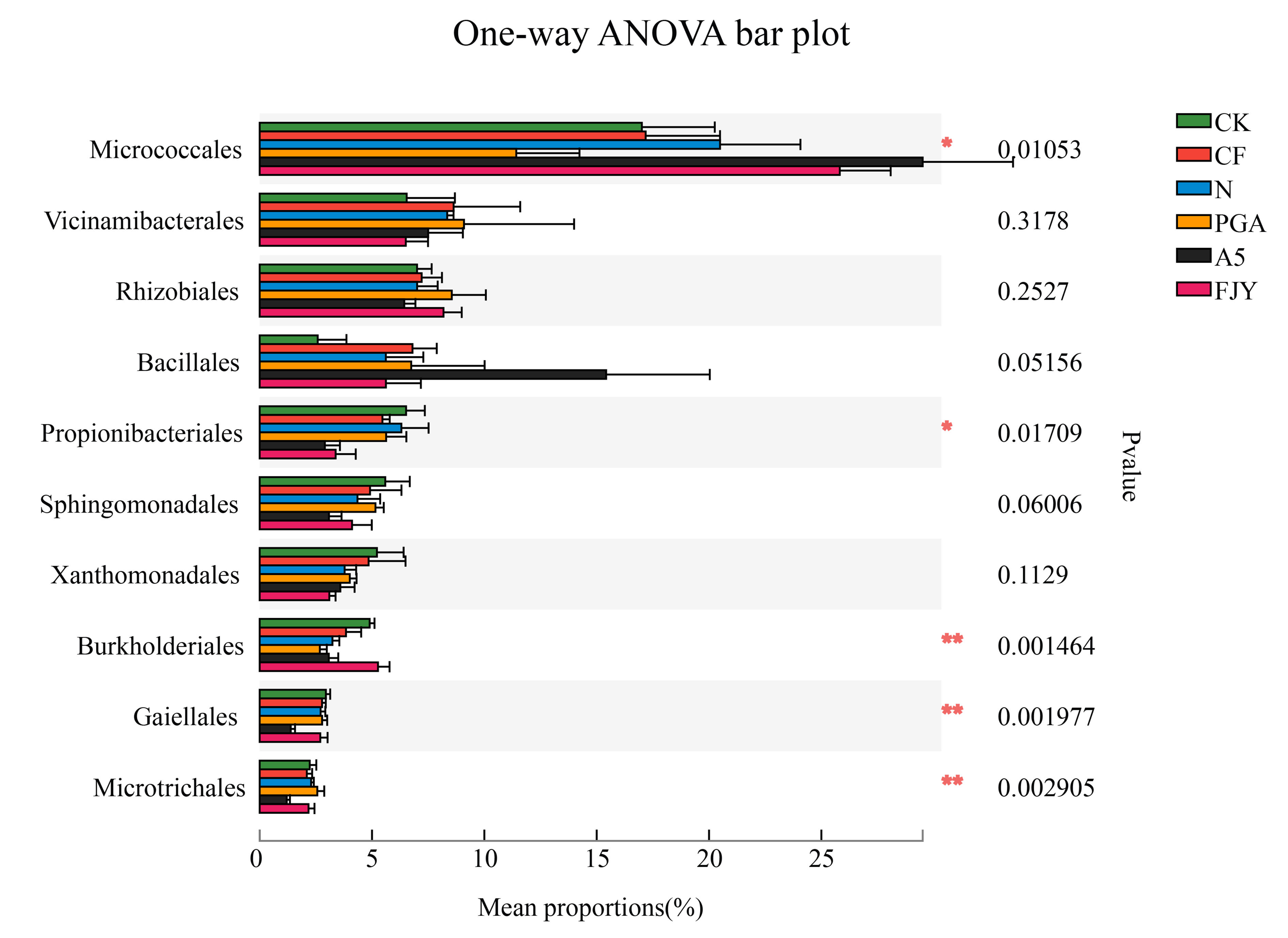

Supplement: Supplementary Figure 1 — One-way ANOVA of the top 10 bacterial taxa with significant differences at the order level. “*” and “**” indicate significant differences (P < 0.05) and extremely significant differences (P < 0.01), respectively. [file Image_1.TIF]

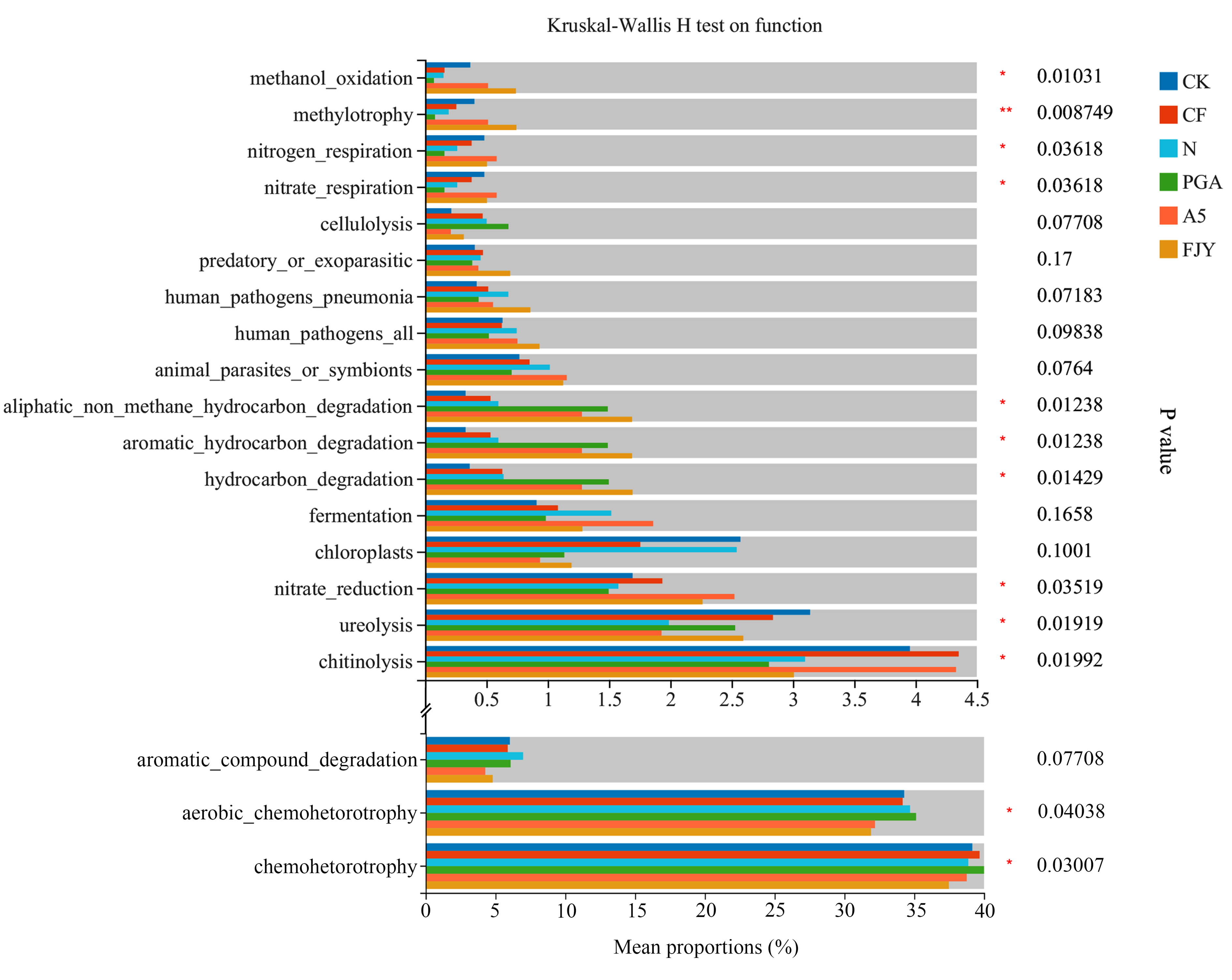

Supplement: Supplementary Figure 2 — Statistically significant difference analysis in rhizosphere soil bacterial functions under different treatments. “*” and “**” indicate significant differences (P < 0.05) and extremely significant differences (P < 0.01), respectively. [file Image_2.TIF]

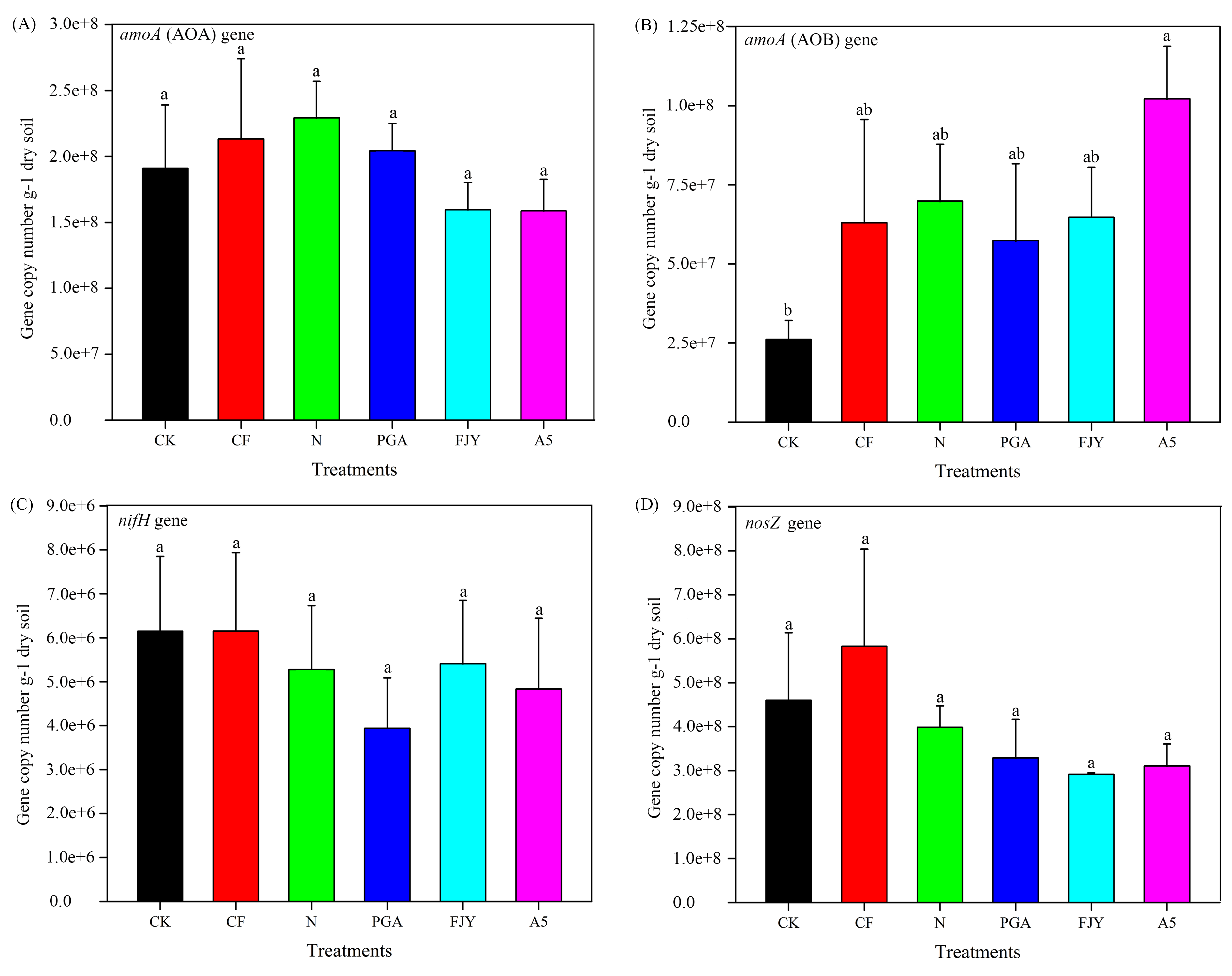

Supplement: Supplementary Figure 3 — qPCR analysis of the expression of N cycling functional genes (amoA [AOA, AOB], nifH, and nosZ). [file Image_3.TIF]
