# Supplementary material for: Effects of Bacillus subtilis A-5 and its fermented γ-polyglutamic acid on the rhizosphere bacterial community of Chinese cabbage
Source: Front Microbiol. 2022 Aug 15;13:954489. doi: 10.3389/fmicb.2022.954489 (PMC9421268; doi:10.3389/fmicb.2022.954489)
Supplement: Supplementary Table 1 — One-way ANOVA of the top 10 bacterial taxa with significant differences at the phylum level (%). [file Table_1.DOCX]

**Table S1** One-way ANOVA of the top 10 bacterial taxa with significant differences at the phylum level (%)

| Phylum | CK | CF | N | PGA | A5 | FJY |
| --- | --- | --- | --- | --- | --- | --- |
| Actinobacteriota | 38.89a | 36.89a | 40.90a | 34.55a | 39.85a | 41.75a |
| Proteobacteria | 27.03a | 25.11a | 23.01a | 25.57a | 20.55a | 25.99a |
| Acidobacteriota | 9.6a | 12.21a | 11.96a | 12.62a | 9.79a | 9.62a |
| Firmicutes | 3.43b | 7.81b | 6.49b | 7.99ab | 12.81a | 6.27b |
| Chloroflexi | 5.8b | 6.21ab | 6.28ab | 8.3a | 4.15b | 4.85b |
| Bacteroidota | 5.74a | 4.11ab | 3.62ab | 3.49b | 4.63ab | 4.24ab |
| Myxococcota | 1.9a | 1.59a | 1.49a | 1.79a | 1.09a | 1.98a |
| Gemmatimonadota | 1.44ab | 1.49a | 1.48a | 1.72a | 0.94b | 1.5a |
| Cyanobacteria | 1.71a | 1.04ab | 1.48ab | 0.72ab | 0.49b | 0.7b |
| Methylomirabilota | 1.37a | 1.03ab | 0.74b | 0.84ab | 0.55b | 0.66b |
