# Supplementary material for: Effects of Bacillus subtilis A-5 and its fermented γ-polyglutamic acid on the rhizosphere bacterial community of Chinese cabbage
Source: Front Microbiol. 2022 Aug 15;13:954489. doi: 10.3389/fmicb.2022.954489 (PMC9421268; doi:10.3389/fmicb.2022.954489)
Supplement: Supplementary Table 2 — One-way ANOVA of the top 10 bacterial taxa with significant differences at the genus level (%). [file Table_2.docx]

**Table S2** One-way ANOVA of the top 10 bacterial taxa with significant differences at the genus level (%)

| Genus | CK | CF | N | PGA | A5 | FJY |
| --- | --- | --- | --- | --- | --- | --- |
| unclassified_f__Micrococcaceae | 11.55b | 11.82b | 14.14ab | 6.08b | 22.22a | 20.98a |
| *Bacillus* | 2.07b | 5.86b | 4.56b | 4.98b | 12.23a | 4.72b |
| norank_f__norank_o__Vicinamibacterales | 3.11a | 4.18a | 4.22a | 5.00a | 3.83a | 3.06a |
| norank_f__Vicinamibacteraceae | 3.15a | 4.10a | 3.79a | 3.62a | 3.37a | 3.17a |
| *Sphingomonas* | 4.22a | 3.81a | 3.28a | 4.07a | 2.40a | 3.37a |
| *Nocardioides* | 3.66a | 3.08abc | 3.56ab | 2.94abc | 1.44c | 1.73bc |
| *Lysobacter* | 2.57a | 2.60a | 1.73a | 1.81a | 2.06a | 1.68a |
| *Gaiella* | 1.96a | 1.74a | 1.74a | 1.82a | 0.95b | 1.83a |
| unclassified_f__Intrasporangiaceae | 1.41a | 1.51a | 1.91a | 1.62a | 1.25a | 1.21a |
| unclassified_f__Nocardioidaceae | 2.04a | 1.46ab | 1.73ab | 1.42ab | 1.01b | 1.08b |
